# Socioeconomic Status and Parental Lifestyle Are Associated With Vascular Phenotype in Children

**DOI:** 10.3389/fpubh.2021.610268

**Published:** 2021-03-26

**Authors:** Sabrina Köchli, Katharina Endes, Julia Grenacher, Lukas Streese, Giulia Lona, Christoph Hauser, Arne Deiseroth, Lukas Zahner, Henner Hanssen

**Affiliations:** Department of Sport, Exercise and Health, Medical Faculty, University of Basel, Basel, Switzerland

**Keywords:** parental lifestyle, socioeconomic status, migration background, arterial stiffness, retinal microcirculation

## Abstract

**Background/Aims:** Socioeconomic barriers and lifestyle conditions affect development of cardiovascular disease in adults, but little is known about the association of parental lifestyle and education with childhood health. We aimed to investigate the association of socioeconomic status (SES), migration background, parental physical activity (PA) and smoking status with micro-and macrovascular health in children.

**Methods:** In 2016/2017, 833 school children (aged 7.2 ± 0.4 years) in Basel (Switzerland) were screened for retinal arteriolar-to-venular ratio (AVR), pulse wave velocity (PWV), SES, migration background and parental PA as well as smoking status.

**Results:** High parental PA levels were associated with a favorable higher AVR (*p* = 0.020) and lower PWV (*p* = 0.035), but not independent of parental smoking status. Children with parents who smoked had a higher PWV [4.39 (4.35–4.42) m/s] compared to children with non-smoking parents [4.32 (4.29–4.34) m/s, *p* = 0.001]. Children of parents with a low household income had a higher PWV [4.36 (4.32–4.41) m/s] compared to children of parents with a high household income [4.30 (4.26–4.34) m/s, *p* = 0.033]. Low parental educational level was associated with a lower AVR [0.86 (0.85–0.88)] compared to children with highly educated parents [AVR:0.88 (0.87–0.88), *p* = 0.007; PWV: 4.33 (4.30–4.35) m/s, *p* = 0.041]. Children with a European background showed a higher AVR [0.88 (0.87–0.88)] compared to non-European children [AVR: (0.86 (0.85–0.87), *p* = 0.034].

**Conclusion:** Parental PA is associated with better macro- and microvascular childhood health. However, the positive association is lost when parental smoking is considered in the analysis. Socioeconomic factors seem to associate with subclinical vascular alterations in children. Primary prevention programs should focus on including parental lifestyle interventions and educational programs to reduce the burden of lifestyle-associated barriers in order to improve cardiovascular health during lifespan.

**Clinical Trial Registration:** ClinicalTrials.gov Exercise and Arterial Modulation in Youth, https://clinicaltrials.gov/ct2/show/NCT02853747, NCT02853747.

## Introduction

In high-income western countries such as Switzerland, cardiovascular (CV) disease is the leading cause of death. CV disease has its origin early in life due to genetic predisposition, environmental influence as well as socioeconomic and lifestyle-associated status. Over the last decades, the assessment of retinal vessel diameters and arterial stiffness by aortic pulse wave velocity (PWV) have become well-established and recommended vascular biomarkers as a surrogate end point for CV risk assessment (1–4). We recently demonstrated that childhood obesity, high blood pressure (BP) and low physical fitness are associated with retinal micro- and macrovascular alterations in young children (5). Children with obesity and elevated BP had higher arterial stiffness and narrower retinal arteriolar diameters. Cardiorespiratory fitness was associated with a favorable higher retinal arteriolar-to-venular ratio (AVR) as well as lower arterial stiffness (5). Environmental conditions such as socioeconomic status (SES), migration background and parental lifestyle seem to play a major role in the development of CV risk factors in children and adolescence. Evidence suggests that the prevalence of CV risk factors is higher in children with low SES. For example, an inverse correlation has previously been described between household income and childhood overweight and obesity (6, 7). The International Study of Childhood Obesity, Lifestyle and the Environment in 12 countries around the world found that parental educational level is also inversely associated with childhood overweight and obesity (8). Parental educational level seems to be contributed to a higher prevalence of childhood obesity (9), elevated BP (10, 11) and sedentary behavior (7, 12). Passive smoking seems to affect development of childhood obesity (13) and vascular health (14, 15). Migration background has also been associated with higher prevalence of children with obesity and physical inactivity (7, 9, 12). Nonetheless, evidence for associations between socioeconomic status, parental smoking and physical activity (PA) behavior with childhood macro- and microvascular health are scarce. This study, for the first time, aimed to investigate the association of SES, migration background and parental lifestyle with large artery stiffness and retinal microvascular health in young school children.

## Method

### Study Design and Participants

This study is part of the large-scale, cross-sectional EXAMIN YOUTH study (16). Children were included if they were between 6 and 8 years old and had an informed consent from their parents to participate. Briefly, children with written informed consent were screened for large artery stiffness and retinal vessel diameters to assess macro-and microvascular health. Anthropometric parameters and blood pressure were determined according to standardized procedures for children. In addition, physical fitness was assessed on a separate day. The medical screening was performed in the morning on-site the regular school setting and children had to remain fastened before anthropometric and CV assessments. Parents were requested to complete a questionnaire on lifestyle behavior, level of education and SES (16). The questionnaires were handed out and re-collected by the teachers. If the questionnaire was not returned to the teachers by the parents, an additional reminder was sent to the parents asking them to return the completed questionnaire. Approval from the Ethics Committee of the University of Basel (EKBB, Basel, No. 258/12) was conferred. The study was designed according to the Guidelines for Good Clinical Practice of the Declaration of Helsinki (17) and the manuscript conforms to The Strengthening the Reporting of Observational Studies in Epidemiology (STROBE) Guidelines (18).

### Measurements

#### Retinal Microcirculation

Retinal vessels were assessed using a fundus camera (Topcon) and an advanced image processing unit (Visualis 3.1, Imedos Systems, Jena, Germany). This technique allows a non-invasive and semi-automated measurement of retinal vessel diameters. The method has been described elsewhere (16). Briefly, two valid images from the retina of the left and the right eye were taken at an angle of 45° with the optic disc in the center. Retinal vessel diameters were estimated to central retinal arteriolar (CRAE) and central retinal venular diameter equivalents (CRVE) applying the Parr-Hubbard formula (19). The AVR was calculated from CRAE and CRVE. CRAE and CRVE were presented in μm. One measuring unit assigns one μm in the Gullstrand‘s normal eye. Reproducibility for retinal vessel analysis is high with an interclass coefficient for CRAE of *r* = 0.94 and a coefficient of variation of about 2% in young children (20).

#### Large Artery Stiffness

PWV serves as a well-established and validated indicator of arterial stiffness (21). The assessment of PWV was performed using the non-invasive and validated oscillometric Mobil-O-Graph Monitor (I.E.M. GmbH, Germany) with integrated ARCSolver software (22–24). PWV was achieved in a sitting position and appropriate small-sized cuffs were settled on the left upper arm. After a resting period of 5 min, a blood pressure measurement was performed, which ensured a calibration with systolic BP. Two measurements of pulse wave analysis followed. After checking every measurement for erroneous values, the mean of two valid measurements was used for further analysis. Data on the validity for use of the Mobil-O-Graph in children has previously been published (5, 25).

#### Anthropometric Parameters and Physical Fitness

Anthropometric measurements and physical fitness assessments have been explained in detail elsewhere (16). Briefly, body height and weight (InBody 170 Biospace device; InBody Co., Seoul, Korea) were measured in light sport clothes without wearing shoes, and BMI was calculated. The InBody device has been validated in school-aged children and correlates strongly with the measurement of dual-energy X-ray absorptiometry (26). BP was assessed five times using an automated oscillograph (Oscillomate, CAS Medical Systems, Branford, CT, USA) after 5 min of rest and based on the recommendations of the American Heart Association. Physical fitness was objectively measured by the validated 20-m shuttle run test (27, 28).

#### Questionnaire

Parents were asked to fill in a questionnaire regarding their SES, migration background and parental lifestyle. Questionnaire items were included from our previous study (7) and translated into the seven most spoken languages in Switzerland.

#### Socioeconomic Status

SES included household income and parental educational level. Household income was determined as low (under CHF 5000/month), medium (CHF 5000-9000/month) and high (over CHF 9000/month) income. Parental educational level referred to the highest school level completed by at least one parent. A low educational level was defined as both parents having/ not having completed compulsory school education, whereas neither of them absolved a vocational training. A medium educational level was determined as at least one parent having completed compulsory school education and vocational training. A high educational level was defined as at least one parent having completed high-school education with or without tertiary education.

#### Migration Background

Children were categorized into children with two-sided non-European migration background (two non-European parents), migrants with one-sided European migration background (only one non-European parent) or without migration background (both parents are European).

#### Parental Lifestyle

Parental lifestyle consisted of parental PA level and smoking status. Parental PA was categorized into three groups. Low PA was determined as both parents never being physically active or no more than once a week. Medium PA was defined as at least one parent being physically active twice a week. High PA was defined as at least one parent being physically active several times per week or on a daily basis. Additionally, parents were asked if they were smoker, or non-smoker.

#### Statistical Analysis

Univariate analysis of covariate (ANCOVA) was used to analyse the association of household income, educational level, parental PA level and smoking status. Different models were fitted to adjust for age and sex as well as household income, educational level and parental lifestyle. Bonferroni *post hoc* testing was conducted to reveal the direction of the results. Additionally, we applied sensitivity analysis to adjust for further potential confounders. For quantitative analysis of parental PA categories and smoking status, bivariate analysis was performed. To indicate the amount of uncertainty the measurement of effect presents 95% confidence intervals (CI) and a two-sided level of significance of *p* = 0.05 denotes statistical significance. All analyses were performed using an up-to-date version of Stata 15 (StataCorp LP, College Station, TX, USA). The sample size of the study was given by the expected large number of children and parents giving their consent and was based on calculations of our previous smaller scale study (7, 20). In this study conducted by Imhof et al., we assumed a moderate effect size for the influence of a physical fitness performance on retinal vessel diameters in children. The sample size calculated with the software *G*-Power using *F*-tests (f = 0.25, power = 0.90% and 5% level of significance) was estimated to be ~290 children in total.

## Results

**Figure 1:**
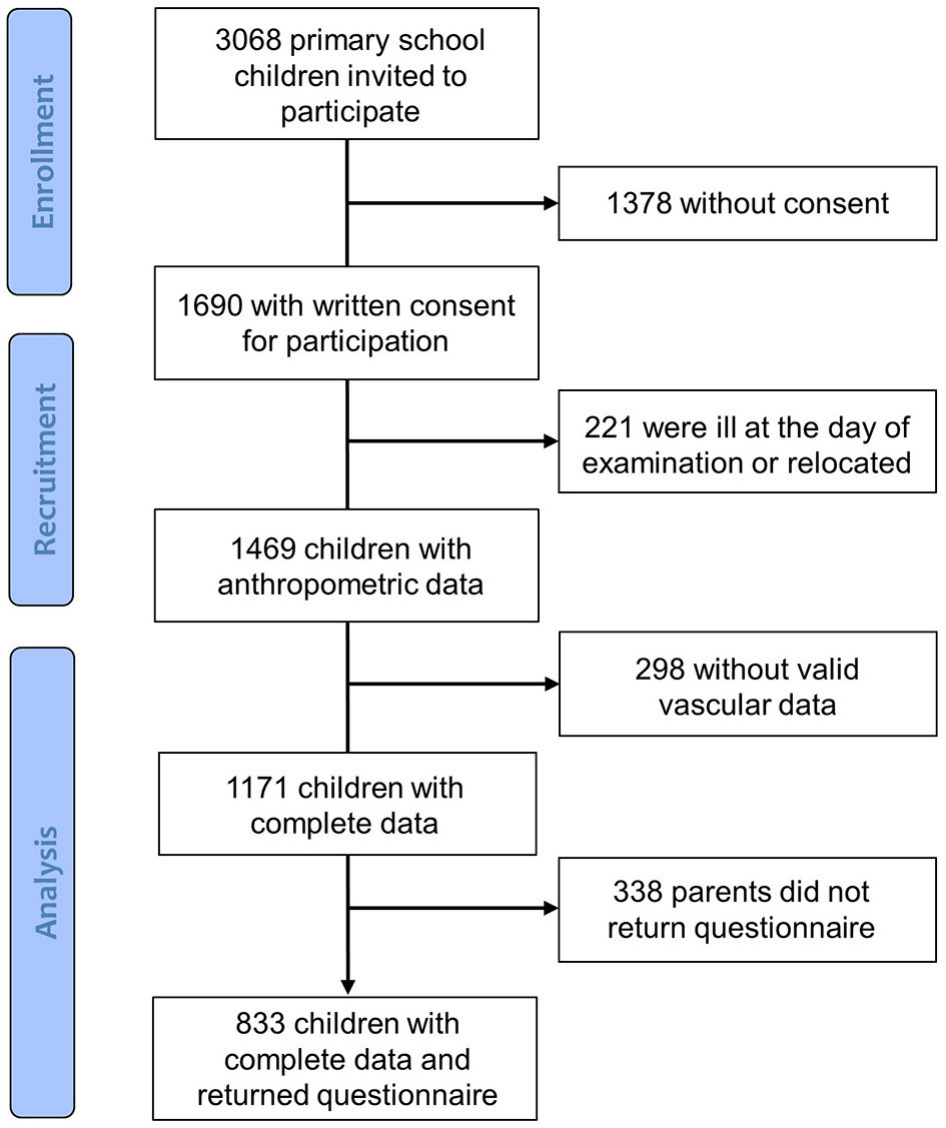
Flow diagram.

Population characteristics are presented in Table 1. In our cohort, 3,068 children received an invitation and informed consent to participate. From 1,690 children with written consent from their parents, 221 children were ill at the day of examination. As can be seen from the flow diagram Figure 1, 833 children eventually had complete data from the medical screening and the returned questionnaires. Age, body weight and height, body fat, BMI and physical fitness data of the 636 excluded children are presented in Supplementary Table 1. Excluded children had lower fitness levels compared to children included in the study.

**Table 1:** Population characteristics.

**Parameter**	***n***	**Mean**	**SD**
Age	1,171	7.2	0.4
Sex			
Female	594		
Male	577		
Height (mm)	1,171	1,244	55
Weight (kg)	1,171	24.6	4.7
BMI (kg/m^2^)	1,171	15.8	2.2
BMI-SDS (kg/m^2^)	1,171	−0.06	1.20
Percentage body fat (%)	1,171	15.3	7.7
Physical fitness (shuttle run stages)	1,171	3.8	1.5
Heart rate (bpm)	1,171	85.6	10.4
Systolic blood pressure (mmHg)	1,171	103.6	7.7
Diastolic blood pressure (mmHg)	1,171	64.1	6.8
Mean arterial blood pressure (mmHg)	1,171	77.3	6.5
CRAE (μm)	1,171	203.0	13.6
CRVE (μm)	1,171	230.3	14.1
AVR	1,171	0.88	0.05
PWV (m/s)	1,171	4.4	0.3

*BMI, body mass index; SDS, standard deviation score; CRAE, central retinal arteriolar equivalent; CRVE, central retinal venular equivalent; AVR, arteriolar-to-venular diameter ratio; PWV, pulse wave velocity; SD, standard deviation*.

### Analysis of Covariance

The results for differences in group-categories are shown in Table 2.

**Table 2:** Retinal vessel diameters and pulse wave velocity in relation to categories of household income, education level, migration background, parental smoking and physical activity behavior.

**Parameter**	***n***	**CRAE (μm)Mean (95% CI)**	***P***	**CRVE (μm)Mean (95% CI)**	***P***	**AVRMean (95% CI)**	***P***	**PWV (m/s)Mean (95% CI)**	***P***
**Household income**			0.185		0.770		0.114		0.002
Low(<CHF 5000/month)	250	203.0(201.3;204.7)		231.4(229.6;233.3)		0.87(0.87;0.88)		4.37(4.33;4.41)	
Medium(CHF5000-9000/month)	279	202.3(200.7;204.0)		230.5(228.8;232.3)		0.87(0.87;0.88)		4.37(4.33;4.41)	
High(>CHF 9000/month)	304	204.4(202.9;205.9)		230.8(229.1;232.4)		0.88(0.87;0.89)		4.29(4.26;4.33)	
**Household income**^**a**^			0.511		0.974		0.661		**0.033**
Low(<CHF 5000/month)	250	203.6(201.6;205.5)		231.1(229.0;233.2)		0.88(0.87;0.89)		4.36(4.32;4.41)	
Medium(CHF5000-9000/month)	279	202.6(200.9;204.3)		230.7(229.0;232.6)		0.87(0.87;0.88)		4.37(4.34;4.41)	
High(>CHF 9000/month)	304	204.0(202.2;205.8)		230.9(229.0;232.8)		0.88(0.87;0.88)		4.30(4.26;4.34)	
**Educational level**			0.141		0.415		0.007		0.041
Low(obligatory school)	83	200.5(197.7;203.4)		230.5(227.5;233.5)		0.86(0.85;0.88)		4.40(4.34;4.47)	
Medium(vocational school)	181	203.2(201.3;205.2)		231.8(229.8;233.8)		0.87(0.86;0.88)		4.37(4.33;4.42)	
High(tertiary education)	569	203.6(202.5;204.7)		230.2(229.1;231.4)		0.88(0.87;0.88)		4.33(4.30;4.35)	
**Educational level**^**b**^			0.165		0.475		0.060		0.420
Low(obligatory school)	83	200.6(197.4;203.8)		230.8(227.4;234.3)		0.86(0.85;0.88)		4.39(4.31;4.46)	
Medium(vocational school)	181	204.0(201.8;206.1)		232.2(229.9;234.5)		0.87(0.86;0.88)		4.33(4.28;4.38)	
High(tertiary education)	569	203.7(202.4;204.9)		230.5(229.2;231.8)		0.88(0.87;0.88)		4.34(4.31;4.37)	
**Migration background**			0.768		0.007		0.019		0.371
European	654	203.0(202.0;204.0)		229.9(228.9;231.0)		0.88(0.87;0.88)		4.35(4.33;4.37)	
One-sided	106	204.0(204.0;203.4)		232.0(229.3;234.6)		0.87(0.86;0.88)		4.36(4.30;4.41)	
Two-sided	73	203.4(200.3;206.4)		235.1(231.9;238.2)		0.86(0.85;0.87)		4.30(4.23;4.37)	
**Migration background**^**c**^			0.874		**0.012**		**0.034**		0.123
European	654	203.2(202.2;204.3)		230.2(229.0;231.3)		0.88(0.87;0.88)		4.35(4.33;4.38)	
One-sided	106	203.6(200.9;206.3)		232.0(229.2;234.9)		0.87(0.86;0.88)		4.35(4.29;4.41)	
Two-sided	73	204.1(200.8;207.4)		235.7(232.1;239.2)		0.86(0.85;0.87)		4.27(4.19;4.34)	
**Physical activity**			0.482		0.481		0.020		0.035
Low (<1/week)	211	202.7(200.8;204.6)		232.0(230.0;233.9)		0.87(0.86;0.87)		4.39(4.35;4.44)	
Medium (1/week)	177	202.5(200.5;204.5)		230.8(228.7;232.9)		0.87(0.86;0.88)		4.32(4.27;4.37)	
High (>1/week)	445	203.8(202.5;205.0)		230.5(229.2;231.8)		0.88(0.87;0.88)		4.33(4.30;4.36)	
**Physical activity**^**d**^			0.573		0.616		0.071		0.098
Low (<1/week)	211	202.8(200.9;204.7)		231.7(229.7;233.7)		0.87(0.86;0.88)		4.38(4.34;4.43)	
Medium (1/week)	177	202.5(200.5;204.5)		230.8(228.7;232.9)		0.87(0.86;0.88)		4.32(4.28;4.37)	
High (>1/week)	445	203.6(202.4;204.9)		230.5(229.2;231.9)		0.88(0.87;0.88)		4.34(4.31;4.36)	
**Parental smoking status**			0.155		0.637		0.013		<0.001
Non-smoker	555	203.5(202.4;2046)		230.4(229.2;231.6)		0.88(0.87;0.88)		4.32(4.29;4.34)	
Smoker	278	202.1(200.6;203.7)		230.9(229.3;232.5)		0.87(0.86;0.88)		4.39(4.35;4.42)	
**Parental smoking status**^**e**^			0.291		0.704		0.057		**0.001**
Non-smoker	555	203.4(202.3;204.5)		230.6(229.4;231.8)		0.88(0.87;0.88)		4.32(4.29;4.34)	
Smoker	278	202.4(200.8;204.0)		231.0(229.3;232.7)		0.87(0.86;0.88)		4.39(4.35;4.42)	

*Data adjusted for age and gender, P-value across lowest and highest category (univariate analysis of covariance)*.

a *Additionally adjusted for educational level*.

b *Additionally adjusted for household income*.

c *Additionally adjusted for household income and educational level*.

d *Additionally adjusted for smoking status*.

e *Additionally adjusted for parental physical activity level*.

*CRAE, central retinal arteriolar equivalent; CRVE, central retinal venular equivalent; AVR, arteriolar-to-venular diameter ratio; PWV, pulse wave velocity; CI, confidence interval; bold values are statistically significant associations after adjustment*.

### Socioeconomic Status

Thirty percentage (*n* = 250) of children were in the group with low, 33% (*n* = 279) with medium, and 37% (*n* = 304) with high household income. Eleven percentage (*n* = 83) were children with low, 22% (*n* = 181) with medium, and 67% (*n* = 569) with highly educated parents. Children with low household income parents had a higher PWV compared to children with high household income parents, also after adjustment for educational level (*p* = 0.033). Retinal vessel diameters were not associated with household income. Lower parental educational level was associated with a lower AVR (*p* = 0.007) and higher PWV (*p* = 0.041), but not independent of household income.

### Migration Background

In our cohort, 9% (*n* = 73) of children had two-sided non-European parents, 13% (*n* = 106) had one-sided non-European parents and in 78% (*n* = 399) of children both parents were European.

Children with a European background showed narrower CRVE (*p* = 0.012) and higher AVR (*p* = 0.034) compared to non-European children, independent of household income and parental education. European migration background was not associated with arterial stiffness.

### Parental Lifestyle

Based on parental PA level, 25% (*n* = 211) were classified as parents with a low PA level, 21% (*n* = 177) as medium and 54% (*n* = 445) as parents with a high PA level. Based on parental smoking status, 67% (*n* = 555) were non-smoking parents.

**Figure 2:**
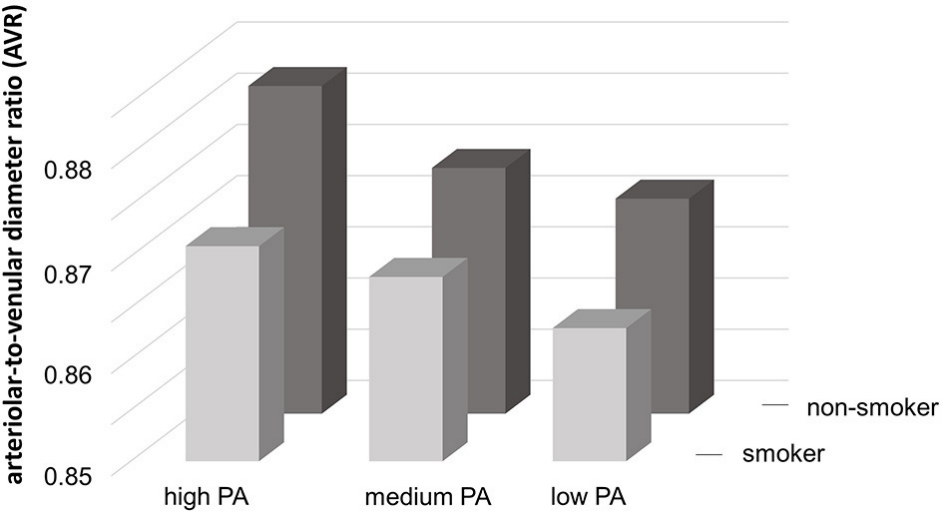
Interrelation between AVR and parental physical activity categories and smoking status. PA, physical activity.

Higher parental PA level was related to higher AVR (*p* = 0.020) and lower PWV (*p* = 0.035), but not after adjustment for parental smoking status. Parental smoking status was associated with a lower AVR (*p* = 0.013), but not independent of parental PA level. Children with smoking parents had a higher PWV compared to children with non-smoking parents (*p* = 0.001), also after adjustment for potential confounders. Bivariate analysis illustrated that microvascular AVR increased according to increasing parental PA level and a non-smoking status (*p* < 0.001) (Figure 2).

### Association With Parental Gender

Mother's and Father's educational level, migration background, smoking and PA behavior are shown separately in Supplementary Table 2. As a main result, children with a mother of high education had a higher AVR (*p* = 0.001) due to wider arterioles, independent of household income. The educational level of the father was not associated with childhood vascular health. Children with a European mother had a higher AVR (<0.001) due to narrower venules (*p* = 0.001) compared to children with a two- or one-sided non-European mother independent of household income and educational level. The migration background of the father was not independently associated with childhood vascular health. In children with high physically active mothers, retinal microvascular AVR was higher (*p* = 0.020) independent of parental smoking status. The PA level of the father was not associated with childhood vascular health. However, smoking status of the father was associated with an unfavorable lower AVR (*p* = 0.044) and a higher PWV (*p* = 0.001) independent of parental PA levels.

## Discussion

Our study demonstrated several detrimental associations of socioeconomic and migration status as well as parental lifestyle with macro- and microvascular health in young children.

The influence of parental lifestyle on vascular phenotype was evident examining parental PA and smoking status with both, retinal vessel diameters and PWV. Children with smoking parents had higher arterial stiffness, independent of parental PA level. The analyses of the interrelation between parental PA categories and smoking status with microvascular AVR demonstrate the additive deleterious effects of parental physical inactivity and smoking on the microvascular phenotype of the children (Figure 2). Children with smoking and low physically active parents showed the lowest AVR in retinal microcirculation.

Parental PA levels and smoking status affect the vascular phenotype in young children. It has previously been shown that parental PA and perceptions of competence are associated with parental support for children's PA and therefore, for children's PA at home (29, 30). This could be one explanation for the association of parental PA level with vascular health in our study. In addition, there is evidence that childhood exposure of environmental tobacco smoke is associated with vascular functional impairments in adulthood (14, 15). In line with our results, a previous study demonstrated that passive smoking is associated to attenuated endothelial dysfunction in 11-year old children (31). Vascular alterations are related to impaired arterial vasodilatation. Arterial dilatation is mediated in large part by the release and bioavailability of nitric oxide. Our findings suggest that the lower AVR and higher arterial stiffness in smoking-exposed children may be due to the interaction of smoke particles with the nitric oxide pathway.

In our cohort of young children, higher household income was independently associated with lower arterial stiffness. The Young Finns study previously reported that childhood SES, defined as annual household income and years of parental education, is inversely associated with PWV in adulthood (32). Previous studies have shown that parental educational level seems to contribute to a higher prevalence of childhood obesity (9), elevated BP (10, 11) and sedentary behavior (7, 12). Lower maternal education has been associated with higher blood pressure in 6 year old children (11). However, no relationship between maternal education and arterial stiffness was found (11). In line with previous findings, parental education was not independently associated with PWV in our cohort. Our results show that high maternal education is related to favorable microvascular health (wider CRAE and higher AVR). Interestingly, maternal SES and lifestyle behavior was primarily associated with vascular health of children. An exception was seen for smoking status of the father which was independently association with micro- and macrovascular alterations of children. In adults, it has been shown that narrower CRAE and wider CRVE as well as higher arterial stiffness are predictors for increased risk of CV disease (1, 33). Therefore, disadvantaged SES may be an important influencing factor for the development of CV disease later in life.

With respect to migration background, European children had narrower CRVE and a higher AVR compared to non-European children. Similar results were found in a previous smaller-sized study on migration status and retinal microcirculation (7). In our cohort, no association of migration background and PWV was observed. There is evidence that migration background is associated with CV risk factors such as childhood obesity and sedentary behavior (7, 9, 12). We recently showed that childhood obesity and elevated blood pressure are related to retinal vessel alterations and arterial stiffness in this cohort of children (5). Based on our findings, we assumed that both micro- and macrovascular beds give separate clinically relevant information on health disadvantaged of children with migration background.

Some limitations have to be discussed. Our cross-sectional study is associative in nature and thus no differentiation in terms of causality can be made. Causal associations of vascular alterations with the development of CV disease in adulthood have to be verified by a longer-term follow up study. Our study was designed in a school setting and 45% of parents did not give consent for participation. Oftentimes parents find it difficult to follow instructions and to fully comprehend the content of the questionnaire and may therefore have decided not to participate (34). The low participation rates in questionnaire-based surveys are common limitations (34, 35). We were not able to compare participants with non-participants with respect to parental lifestyle and SES. However, children who did not participate in the medical screening and did not have a completed questionnaire had a similar BMI, but a lower physical fitness compared to those participating. The risk of a selection bias was therefore considered to be moderate. The oscillometric measurement with the Mobil-O-Graph device to estimate PWV is based on a calibration with systolic blood pressure. The assessment is thus associated with level of current systolic blood pressure. The assessment of PWV in large cohort of school children has been shown to be a validated approach (5). We refrained from additionally adjusting for systolic blood pressure in our statistical models to avoid over-adjustment. Our findings are related to a predominant Caucasian population with a small percentage of other ethnical groups. We have therefore chosen to characterize our population by looking at non-European migration background. Future studies have to investigate the association of SES, migration background and parental lifestyle with vascular health in other ethnic groups and populations from different countries. One strength of our study is the large sample size and the limited age range of young children. During childhood, mirco- and macrovascular function and structure continuously develop, and especially during puberty, age adaptations occur rapidly. Investigating different vascular beds in a large sample of children at the same age therefore reduces a developmental impact of our findings. Future studies may still investigate the association of SES, migration background and parental lifestyle with vascular health in a wider age range from infancy to adolescence to account for the dynamic pattern of small and large vessel development during childhood.

## Conclusion

Parental PA has been found to be associated with better micro- and macrovascular health but not independent of parental smoking status. It appears that parental smoking may mitigate the positive association of parental PA on childhood vascular health. Parental smoking was itself independently associated with impaired large artery stiffness in children. Cessation of parental smoking therefore seems key to improve childhood vascular health. Prospective follow-up studies will have to prove if cessation of parental smoking can improve CV outcome in offspring long-term. SES and migration background also affect micro-and macrovascular impairments early in life. Primary treatment strategies will have to address reduction of socioeconomic barriers. Family-based interventions targeting cessation of parental smoking, increase of parental PA, and reduction of socioeconomic barriers may be effective means to counteract the development of CV risk and disease in offspring and during lifespan.

## Data Availability Statement

The raw data supporting the conclusions of this article will be made available by the authors, without undue reservation.

## Ethics Statement

The studies involving human participants were reviewed and approved by Ethics Committee of the University of Basel (EKBB, Basel, No. 258/12). Written informed consent to participate in this study was provided by the participants' legal guardian/next of kin.

## Author Contributions

SK planned and conducted the study, collected data, performed the statistical analysis, prepared and revised the manuscript. KE designed the study and revised the manuscript. JG collected data and revised the manuscript. LS, GL, and CH interpreted data and revised the manuscript. LZ designed the study and revised the manuscript. HH conceptualized and designed the study, discussed the statistical analysis, prepared and critically reviewed the manuscript. All authors approved the final manuscript as submitted and agreed to be accountable for all aspects of the work.

## Conflict of Interest

The authors declare that the research was conducted in the absence of any commercial or financial relationships that could be construed as a potential conflict of interest.
