# Supplementary material for: Socioeconomic Status and Parental Lifestyle Are Associated With Vascular Phenotype in Children
Source: Front Public Health. 2021 Mar 26;9:610268. doi: 10.3389/fpubh.2021.610268 (PMC8032988; doi:10.3389/fpubh.2021.610268)
Supplement: Supplementary file 1 [file Table_1.DOCX]

| **Table S1.** Population characteristics of the main population as compared to children excluded. | | | | | |
| --- | --- | --- | --- | --- | --- |
| **Parameter** | **Main population**  **Mean±SD (range)** | **n** | **Excluded children**  **Mean±SD**  **(range)** | **n** | **p** |
| Age (years) | 7.2±0.4  (6.2;8.3) | 833 | 7.2±0.4  (6.2;8.3) | 636 | 0.821 |
| Height (cm) | 124.4±5.4  (109.0;141.0) | 833 | 124.4±6.0  (106.9;144.0) | 636 | 0.965 |
| Weight (kg) | 24.5±4.6  (16.2;48.7) | 833 | 24.8±5.1  (13.9;48.7) | 636 | 0.236 |
| BMI (kg/m^2^) | 15.8±2.1  (12.1;26.3) | 833 | 15.9±2.3  (10.0;26.1) | 636 | 0.155 |
| Physical fitness  (shuttle run stages) | 3.8±1.5  (0.5;8.5) | 833 | 3.6±1.5  (0.5;8.5) | 636 | 0.016 |

BMI, body mass index; SD, standard deviation
