# Supplementary material for: Socioeconomic Status and Parental Lifestyle Are Associated With Vascular Phenotype in Children
Source: Front Public Health. 2021 Mar 26;9:610268. doi: 10.3389/fpubh.2021.610268 (PMC8032988; doi:10.3389/fpubh.2021.610268)
Supplement: Supplementary file 2 [file Table_2.DOCX]

| **Table S2.**  Retinal vessel diameters and pulse wave velocity in relation to parental gender-related education level, migration background, smoking and physical activity behaviour . | | | | | | | | | |
| --- | --- | --- | --- | --- | --- | --- | --- | --- | --- |
| **Parameter** | **n** | **CRAE (µm)**  **Mean (95% CI)** | **P** | **CRVE (µm)**  **Mean (95% CI)** | **P** | **AVR (mu)**  **Mean (95% CI)** | **P** | **PWV (m/s)**  **Mean (95% CI)** | **P** |
| **Educational level mother**  Low  (obligatory school)  Medium  (vocational school)  High  (tertiary education) | 146  212  474 | 200.4  (198.4;202.5)  203.8  (202.0;205.6)  203.8  (202.6;205.0) | 0.017 | 231.1  (228.9;233.4)  231.1  (229.2;233.0)  230.3  (229.1;231.6) | 0.702 | 0.86  (0.85;0.87)  0.87  (0.87;0.88)  0.88  (0.88;0.89) | < 0.001 | 4.39  (4.34;4.44)  4.35  (4.31;4.39)  4.33  (4.30;4.35) | 0.051 |
| **Educational level mother^a^**  Low  (obligatory school)  Medium  (vocational school)  High  (tertiary education) | 146  212  474 | 200.3  (197.9;202.7)  204.2  (202.3;206.1)  203.9  (202.5;205.2) | **0.022** | 231.1  (228.5;233.8)  231.6  (229.5;233.6)  230.6  (229.1;232.0) | 0.765 | 0.86  (0.85;0.87)  0.88  (0.87;0.88)  0.88  (0.87;0.89) | **0.001** | 4.37  (4.32;4.43)  4.33  (4.29;4.37)  4.34  (4.31;4.37) | 0.386 |
| **Educational level father**  Low  (obligatory school)  Medium  (vocational school)  High  (tertiary education) | 134  216  483 | 201.8  (199.6;204.0  203.3  (201.5;205.1)  203.5  (202.4;204.7) | 0.414 | 230.8  (228.5;233.1)  231.0  (229.1;232.9  230.4  (229.2;231.6) | 0.872 | 0.87  (0.86;0.88)  0.87  (0.87;0.88)  0.88  (0.87;0.88) | 0.0882 | 4.37  (4.32;4.42)  4.37  (4.33;4.41)  4.33  (4.30;4.35) | 0.118 |
| **Educational level father^a^**  Low  (obligatory school)  Medium  (vocational school)  High  (tertiary education) | 134  216  483 | 202.0  (199.5;204.6)  203.9  (202.0;205.9)  203.5  (202.2;204.9) | 0.460 | 231.0  (228.3;233.7)  231.4  (229.3;233.5)  230.67  (229.2;232.1) | 0.860 | 0.87  (0.86;0.88)  0.87  (0.87;0.88)  0.88  (0.87;0.88) | 0.257 | 4.35  (4.29;4.41)  4.34  (4.29;4.38)  4.34  (4.31;4.37) | 0.918 |
| **Migration background mother**  European  Non-European | 700  131 | 203.2  (202.3;204.2)  203.0  (200.8;205.3) | 0.881 | 230.0  (229.0;231.0)  234.1  (231.8;236.5) | 0.002 | 0.88  (0.87;0.88)  0.86  (0.85;0.87) | <0.001 | 4.35  (4.32;4.37)  4.33  (4.29;4.38) | 0.544 |
| **Migration background mother^b^**  European  Non-European | 700  131 | 203.4  (202.3;204.4)  203.4  (201.0;205.9) | 0.949 | 230.1  (229.0;231.2)  234.8  (232.2;237.4) | **0.001** | 0.88  (0.87;0.88)  0.86  (0.85;0.87) | **<0.001** | 4.35  (4.32;4.37)  4.31  (4.26;4.37) | 0.238 |
| **Migration background father**  European  Non-European | 714  119 | 203.0  (202.0;204.0)  204.3  (201.9;206.7) | 0.319 | 230.2  (229.2;231.2)  233.3  (230.8;235.8) | 0.025 | 0.88  (0.87;0.88)  0.87  (0.86;0.88) | 0.252 | 4.35  (4.33;4.37)  4.32  (4.26;4.37) | 0.275 |
| **Migration background father^b^**  European  Non-European | 714  119 | 203.2  (202.2;204.0)  204.4  (201.8;207.0) | 0.395 | 230.5  (229.4;231.6)  233.3  (230.5;236.1) | 0.070 | 0.88  (0.87;0.88)  0.87  (0.86;0.88) | 0.306 | 4.35  (4.33;4.37)  4.29  (4.23;4.35) | 0.062 |
| **Physical activity mother**  Low (< 1/week)  Medium (1/week)  High (> 1/week) | 337  220  276 | 203.0  (201.6;204.4)  203.5  (201.8;205.3)  203.3  (201.7;204.8) | 0.902 | 232.1  (230.6;233.6)  231.0  (229.1;232.8)  229.4  (227.8;231.0) | 0.060 | 0.87  (0.86;0.87)  0.88  (0.87;0.88)  0.88  (0.88;0.89) | 0.007 | 4.37  (4.34;4.41)  4.31  (4.27;4.35)  4.33  (4.30;4.37) | 0.040 |
| **Physical activity mother^c^**  Low (< 1/week)  Medium (1/week)  High (> 1/week) | 337  220  276 | 203.0  (201.5;204.4)  203.4  (201.6;205.2)  203.3  (201.7;204.9) | 0.918 | 231.8  (230.2;233.3)  231.2  (229.4;233.1)  229.4  (227.7;231.0) | 0.105 | 0.87  (0.86;0.88)  0.87  (0.87;0.88)  0.88  (0.88;0.89) | **0.020** | 4.37  (4.33;4.40)  4.31  (4.27;4.35)  4.34  (4.30;4.38) | 0.085 |
| **Physical activity father**  Low (< 1/week)  Medium (1/week)  High (> 1/week) | 332  171  330 | 202.5  (201.0;203.9)  202.6  (200.6;204.6)  204.4  (202.9;205.8) | 0.143 | 230.6  (229.1;232.1)  230.2  (228.1;232.9)  231.4  (229.9;232.9) | 0.628 | 0.87  (0.87;0.88)  0.87  (0.87;0.88)  0.88  (0.87;0.88) | 0.086 | 4.37  (4.34;4.41)  4.32  (4.27;4.36)  4.33  (4.30;4.36) | 0.068 |
| **Physical activity father^c^**  Low (< 1/week)  Medium (1/week)  High (> 1/week) | 332  171  330 | 202.3  (200.8;204.8)  202.8  (200.8;204.8)  204.2  (202.8;205.7) | 0.184 | 230.2  (228.6;231.7)  230.4  (228.3;232.6)  231.4  (229.9;232.9) | 0.523 | 0.87  (0.87;0.88)  0.88  (0.87;0.88)  0.88  (0.87;0.88) | 0.386 | 4.37  (4.34;4.40)  4.32  (4.27;4.36)  4.33  (4.30;4.36) | 0.124 |
| **Smoking status mother**  Non-smoker  Smoker | 686  147 | 202.9  (202.0;203.9)  203.5  (201.4;205.5) | 0.659 | 230.4  (229.3;231.4)  231.6  (229.4;233.8) | 0.310 | 0.88  (0.87;0.88)  0.87  (0.87;0.88) | 0.655 | 4.33  (4.31;4.36)  4.37  (4.32;4.41) | 0.219 |
| **Smoking status mother^d^**  Non-smoker  Smoker | 686  147 | 203.0  (202.0;203.9)  203.6  (201.5;205.7) | 0.602 | 230.6  (229.6;231.6)  231.5  (229.3;233.7) | 0.449 | 0.87  (0.87;0.88)  0.87  (0.87;0.88) | 0.978 | 4.33  (4.31;4.36)  4.37  (4.32;4.41) | 0.247 |
| **Smoking status father**  Non-smoker  Smoker | 605  227 | 203.7  (202.7;204.8)  201.3  (199.6;203.0) | 0.016 | 230.7  (229.6;231.8)  230.3  (228.5;232.1) | 0.702 | 0.88  (0.87;0.88)  0.87  (0.86;0.87) | 0.007 | 4.31  (4.30;4.34)  4.40  (4.36;4.44) | <0.001 |
| **Smoking status father^d^**  Non-smoker  Smoker | 605  227 | 203.7  (202.6;204.7)  201.7  (199.9;203.4) | 0.059 | 230.9  (229.8;232.1)  230.4  (228.6;232.3) | 0.662 | 0.88  (0.87;0.88)  0.87  (0.86;0.88) | **0.044** | 4.32  (4.30;4.34)  4.39  (4.36;4.43) | **0.001** |

Data adjusted for age and gender, P value across lowest and highest category (univariate analysis of covariance)

^a^ Additionally adjusted for household income

^b^ Additionally adjusted for household income and educational level

^c^ Additionally adjusted for smoking status

^d^ Additionally adjusted for parental physical activity level

CRAE, central retinal arteriolar equivalent; CRVE, central retinal venular equivalent;

AVR, arteriolar-to-venular diameter ratio; PWV, pulse wave velocity; CI, confidence interval
